# Aspects épidémiologiques et bactériologiques des infections du site opératoire (ISO) dans les services de chirurgie à l’Hôpital National de Niamey (HNN)

**DOI:** 10.11604/pamj.2018.31.33.15921

**Published:** 2018-09-14

**Authors:** Ousmane Abdoulaye, Mahaman Laouali Harouna Amadou, Oumarou Amadou, Ousseini Adakal, Harouna Magagi Larwanou, Laouali Boubou, Djimraou Oumarou, Moussa Abdoulaye, Saidou Mamadou

**Affiliations:** 1 Faculté de Sciences de la Santé, Université Dan Dicko Dan Koulodo de Maradi, Service de Biologie, Centre Hospitalier Régional de Maradi, Niger; 2 Faculté de Sciences de la Santé, Université Dan DickoDanKoulodo de Maradi, Service des Maladies Infectieuses, Centre Hospitalier Régional de Maradi, Niger; 3 Faculté de Sciences de la Santé, Université Dan DickoDanKoulodo de Maradi, Service de Chirurgie, Centre Hospitalier Régional de Maradi, Niger; 4 Service de Biologie, Hôpital National de Niamey, Niger; 5 Faculté de Sciences de la Santé, Laboratoire de Bactériologie Virologie, Université Abdou Moumouni de Niamey, Niger

**Keywords:** Infection du site opératoire, bactéries, résistance aux antibiotiques, Niger, Surgical site infections, bacteria, antibiotic resistance, Niger

## Abstract

Ce travail à consister à étudier les aspects épidémiologiques et bactériologiques des souches bactériennes isolées au cours des infections du site opératoire (ISO) à l'Hôpital National de Niamey. Nous avions mené une étude rétrospective, et descriptive sur une période de 24 mois. Toutes les souches isolées à partir de prélèvements bactériologiques effectués chez les patients présentant une infection du site opératoire ont été identifiées et testés aux antibiotiques selon les méthodes classiques conventionnelles. Les analyses bactériologiques ont permis d'isoler 126 souches bactériennes avec une prédominance de *S.aureus* (n=39, 31%) suivi d'*Escherichia coli* (n = 29, 23%) et de *Pseudomonas aeruginosa* (n=12, 9,5%). Les souches d'*Escherichia coli* étaient sensibles à 100% à l'imipenème. Elles ont montré des résistances marquées à l'ampicilline, l'amoxicilline, l'acide-clavulanique et la ticarcilline. Elles présentaient des résistances variables aux aminosides (62% à la gentamycine, et 78% à l'amikacine), et aux fluoroquinolones (acide nalidixique 74%, pefloxacine 33%, l'ofloxacine 69%, ciprofloxacine 61%). L'ensemble des isolats d'entérobactéries étaient sensibles à l'imipénème. Les souches de *S.aureus* avaient montré des résistances à la Pénicilline G (88,6%) et à l'oxacilline (83%). Elles avaient montré aussi des résistances de 37% et 57% respectivement à la vancomycine et teicoplanine. Par contre, elles étaient sensibles à la lincomycine et aux aminosides testés. Compte tenu de ces résultats, nous pensons qu'il faudra améliorer les protocoles d'antibioprophylaxie et d'antibiothérapie probabiliste dans les services chirurgicaux. Aussi, mener des études périodiques de surveillances des ISO.

## Abstract (en)

This study aims to evaluate the epidemiological and bacteriological features of bacterial strains isolated from surgical site infections (ISO) at the Niamey National Hospital. We conducted a retrospective, descriptive study over a period of 24 months. All strains isolated from bacteriological samplings from patients with a surgical site infection have been identified and tested for antibiotic sensitivity according to conventional methods. The bacteriological analysis allowed the isolation of 126 bacterial strains with a predominance of S.aureus (n=39, 31%) followed by Escherichia coli (n=29, 23%) and Pseudomonas aeruginosa (n=12, 9.5%). The strains of Escherichia coli were 100% sensitive to imipenem. They showed marked ampicillin, amoxicillin, clavulanic acid and ticarcillin resistance. They had variable resistance to aminoglycoside antibiotics (62% to gentamycin, and 78% to amikacin) and to fluoroquinolones (nalidixic acid 74%, pefloxacine 33%, ofloxacin 69%, ciprofloxacin 61%). All enterobacterial isolates were sensitive to imipenem. The strains of S.aureus showed resistance to penicillin G (88.6%) and oxacillin (83%). They also showed resistance to vancomycin and teicoplanin (37% and 57% respectively). By contrast, they were sensitive to lincomycin and aminoglycoside antibiotics tested. In the light of these results, we believe that it will be necessary to improve prophylaxis protocol and probabilistic antibiotic therapy in the Surgical Division and to conduct periodic surveillance studies of the ISO.

## Introduction

Les infections du site opératoire (ISO) incluent toute infection sur le site opéré, survenant dans les 30 jours suivant l'intervention ou dans l'année s'il y a eu mise en place d'un implant ou d'une prothèse [1]. Ils constituent un véritable problème de santé publique en Afrique avec une incidence qui varie de 6,8% à 26% [2-4]. Au Niger, peu d'études ont été réalisées sur la fréquence des ISO. Afin de mieux cerner le problème, réduire les dépenses en santé, éviter l'émergence des bactéries multi-résistantes, il est nécessaire de recourir à une surveillance épidémiologique. C'est pourquoi nous nous sommes fixés comme objectifs d'étudier les aspects épidémiologiques des souches bactériennes isolées au cours des ISO et d'évaluer leur profil de résistance aux antibiotiques à l'Hôpital National de Niamey.

## Méthodes

Il s'agissait d'une étude rétrospective, analytique et descriptive réalisée dans les différents services de chirurgie de l'Hôpital National de Niamey. La population d'étude était constituée de patients opérés et hospitalisés au moins 72 heures dans les services de chirurgie du 1er janvier 2015 au 31 Décembre 2016 présentant des suspicions d'ISO. Chez chaque patient, un prélèvement de pus a été effectué par écouvillonnage ou par ponction à l'aide d'une seringue stérile à usage unique. Ces prélèvements ont été acheminés au laboratoire pour leur exploitation en respectant les bonnes pratiques de laboratoire. Les échantillons obtenus étaient traités selon la procédure en vigueur dans le laboratoire. L'examen macroscopique a permis de noter la couleur, la consistance et l'odeur. L'examen microscopique a permis de réaliser une étude cytologique quantitative et qualitative après coloration au May GreenwaldGiemsa et d'apprécier les bactéries au GRAM positif ou négatif. La mise en culture a été réalisée sur gélose chocolat (GC) + Polyvitex incubée sous cloche. L'identification des microorganismes isolés a été réalisée par les méthodes de bactériologie classique (morphologie et mobilité à l'état frais, coloration de Gram, recherche d'une oxydase, d'une catalase ainsi que les autres caractères biochimiques). L'étude de la sensibilité aux antibiotiques a été réalisée par la méthode de diffusion en gélose. Ainsi, les variables étudiées sont les suivantes: âge, sexe, type de prélèvement, germes identifiés, antibiotiques testés. Tous les patients ayant des dossiers incomplets ont été exclus de cette étude.

## Résultats

Du 1er Janvier 2015 au 31 Décembre 2016, 167 prélèvements provenant des services chirurgicaux ont été reçus et analysés au laboratoire de bactériologie de l'HNN pour suspicions d'ISO. Durant cette période 6740 patients ont été opérés soit une incidence de 2,47%. La population d'étude était constituée de 128 hommes (76,66%) et 39 femmes (23,24%) soit une sex-ratio de 3,3. L'âge moyen était de 31,16 ans avec des extrêmes de 20 mois et 80 ans. L'analyse des 167 prélèvements a donné lieu à 118 cultures positives avec 110 cultures monomicrobiennes et 8 cultures polymicrobiennes à 2 germes, soit un total de 126 souches bactériennes isolées. Au total, 10 genres bactériens ont été identifiés avec 13 espèces (Tableau 1). Les germes identifiés en fonction des services de chirurgie, sont représentés dans le Tableau 2. Le taux de résistance des bactéries Gram négatif et Gram positif est résumé dans le Tableau 3.

**Tableau 1:** Répartition des échantillons selon les germes identifiés

Germes	Effectifs	%
*Acinetobacterbaumannii*	3	2,4
*Citrobacterfreundii*	2	1,6
*Esherichia coli*	**29**	23,0
*Enterobacteraerogenes*	7	5,6
*Enterobactercloacae*	5	4,0
*Klebsiellaoxytoca*	6	4,8
*Klebsiella pneumoniae*	10	7,9
*Proteus mirabilis*	9	7,1
*Pseudomonas aeruginosa*	12	9,5
*Staphylococcus aureus*	**39**	31,0
*Serratiamarcescens*	2	1,6
*Streptococcus pneumoniae*	1	0,8
*Streptococcus sp*	1	0,8
**TOTAL**	126	100

**Tableau 2:** Germes identifiés en fonction des services de chirurgie

Germes	Chirurgie Urologie	Chirurgie digestive	Chirurgie Traumatologie	Neurochirurgie	Total
*Acinetobacterbaumannii*	0	3	0	0	3
*Citrobacterfreundii*	0	0	1	1	2
*Esherichia coli*	0	26	2	1	29
*Enterobacteraerogenes*	0	7	0	0	7
*Enterobactercloacae*	0	5	0	0	5
*Klebsiellaoxytoca*	0	6	0	0	6
*Klebsiella pneumoniae*	0	9	1	0	10
*Proteus mirabilis*	0	6	3	0	9
*Pseudomonas aeruginosa*	1	9	1	1	12
*Staphylococcus aureus*	1	26	9	3	39
*Serratiamarcescens*	0	1	1	0	2
*Streptococcus pneumoniae*	1	0	0	0	1
*Streptococcus sp*	0	1	0	0	1
**Total**	3	99	18	6	126
**%**	**2%**	**79%**	**14%**	**5%**	**100%**

**Tableau 3:** Profil de sensibilité des bactéries isolées aux antibiotiques

Antibiotiques	*S aureus* (N=39)		*Esherichiacoli* (N=29)		*Paeruginosa* (N=12)		*Kpneumonea* (N=10)		*Proteus mirabilis* (N=09)	
	N(R/ST)	%R	(R/ST)	%	N(R/ST)	%	N(R/ST)	%(R)	N(R/ST)	%(R)
Penicilline G	31/35	88,6	-	-	-	-	-	-	-	-
Ampicilline	0/1	0	8/8	100	-	-	4/4	100	1/1	100
Amoxiciciline	-	-	28/28	100	-	-	9/9	100	9/9	100
Acide clavulanique	-	-	21/21	100	-	-	9/10	80	8/8	100
Ticarcilline	-	-	5/5	100	5/7	71,4	2/2	100	2/2	100
Co-ticarcilline	-	-	-	-	-	-	-	-	-	-
Oxacilline	30/36	83	-	-	-	-	-	-	-	-
Imipeneme	-	-	0/27	0	0/10	0	0/9	0	0/6	0
Peperacillinetazobactam	-	-	14/22	63,63	4/11	36,36	3/7	42,85	1/7	14,28
Cefixime	-	-	18/20	90	4/5	75	6/6	100	3/5	60
Cefalotine	-	-	5/5	100	0/1	0	-	-	1/1	100
Cefoxitine	-	-	4/5	80	-	-	1/1	100	1/1	100
Cefotaxime	-	-	15/17	88,23	4/4	100	3/7	75	1/6	16,6
Ceftriaxone	-	-	23/27	85,18	6/6	100	7/8	87,5	4/8	50
Ceftazidine	-	-	21/24	91,30	9/10	90	7/7	100	4/8	50
Cefepime	-	-	6/6	100	0/1	0	-	-	3/3	100
Rifampicine	2/26	7,69	-	-	-	-	-	-	-	-
Acide fusidique	2/21	9,52	-	-	-	-	-	-	-	-
Fosfomycine	0/1	0	-	-	-	-	-	-	-	-
Acide nalidixique	-	-	17/23	73,91	6/7	85,71	6/6	100	3/7	42,85
Ofloxacine	0/2	0	1/3	33,33	-	-	-	-	-	-
Norfloxacine	-	-	1/1	100	-	-	0/1	0	-	-
Ciprofloxacine	13/33	39,39	11/18	61,11	6/11	54,54	6/7	85,71	3/8	37,5
Vancoùmycine	3/8	37,5	-	-	-	-	-	-	-	-
Teicoplanine	4/7	57,14	-	-	-	-	-	-	-	-
Kanamycine	0/1	0	-	-	-	-	-	-	-	-
Gentamycine	6/28	21,42	8/21	38,09	4/11	36,36	2/5	40	2/6	33,33
Amikacine	0/9	0	6/23	26,08	1/10	10	1/7	14,28	0/7	0
Erythromycine	13/31	41,93	-	-	-	-	-	-	-	-
Lincomycine	0/5	0	-	-	-	-	-	-	-	-
Tetramycine	15/34	44,11	-	-	-	-	-	-	-	-
Colistine	-	-	4/21	19,04	0/7	0	1/4	20	3/4	75
Cotrimoxazaole	-	-	8/9	88,88	-	-	3/4	75	1/1	100

## Discussion

La contamination du site opératoire survient le plus souvent au cours de la période opératoire, soit à partir de la flore du patient présent avant l'incision, soit à partir de la flore du personnel, soit à partir des solutions antiseptiques ou d'instruments contaminés [3]. En dépit des progrès réalisés dans le domaine de la chirurgie (niveau d'asepsie, connaissance parfaite des ISO, antibioprophylaxie adaptée), les ISO restent une cause majeure de morbi-mortalité post-opératoire. Ces infections touchent 0,76 % des patients en France [5], 16% au Maroc [6]. Dans les pays en voies de développement comme le Niger, la prévalence des ISO représentent respectivement 7,3% au Bénin [7], 11% au Togo [4], 18% en République Centrafricaine [8] et 23,35% au Burkina Faso [9]. Selon une méta-analyse en Afrique sub-saharienne, l'incidence des ISO variaient de 6,8% à 26% avec une prédominance en chirurgie générale [2]. La survenue des ISO dépend du type de chirurgie mais également du niveau de contamination du site opératoire. Dans notre étude, beaucoup plus de germes étaient retrouvés en chirurgie digestive (78,5 %) que dans les autres services chirurgicaux. Dans notre étude, l'âge moyen des patients atteints d'ISO est de 31,16 avec une prédominance masculine. Kanassoua retrouvait la même prédominance masculine sur une tranche d'âge de 19-30 ans [4]. Au Maroc, Kaabachi avait rapporté une prédominance masculine dans son étude sur l'incidence des ISO en chirurgie orthopédique pédiatrique [10]. Les ISO ont une étiologie bactérienne. La recherche des germes responsables a donné lieu à 118 cultures positives soit 71% avec 110 cultures monomicrobiennes et 8 cultures polymicrobiennes à 2 germes. Au total de 126 souches bactériennes ont été isolées. Ces résultats sont inférieurs à ceux trouvés par Ouédraogo en 2011 avec 84,8% au Burkina Faso [9].

En Côte D'Ivoire, Faye-Kette H a rapporté un rendement similaire de 70% de culture positive [3]. Cela confirme bien que la culture demeure plus sensible que la microscopie dans le diagnostic biologique des ISO, surtout dans les prélèvements des pus. A l'issue de notre étude, l'espèce *S.aureus* était largement majoritaire (31%) suivie de *E.coli* (23%) et de *Pseudomonas aeruginosa* (9,5%). Cette distribution était similaire de celle rapportée dans des études menées dans plusieurs pays d'Afrique [4, 11-13]. Par contre, plusieurs auteurs ont rapporté une distribution différente. C'est ainsi qu'en Côte d'Ivoire, Faye-Kette H avaient rapporté une nette prédominance de *Pseudomonas aeruginosa* [3] alors qu'à Madagascar, une étude rapportait une fréquence d*'Acinetobacterbamannii* de 26,6% par Randriambololona VH *et al* en 2007 [14]. Ces différences s'expliqueraient par le fait que ces germes sont responsables d'hospitalisme infectieux ou infections nosocomiales [15]. L'étude du profil de sensibilité aux antibiotiques a montré que la plupart des bactéries isolées étaient résistantes aux antibiotiques couramment utilisés. Les souches de *S.aureus* ont montré des résistances aux bétalactamines. Par contre les aminosides et la vancomycine gardent une certaine efficacité. Les souches d'entérobactéries dans leur majorité présentaient des taux de résistance très élevés aux bétalactamines. Cependant, elles étaient restées sensibles dans une moindre mesure aux fluoroquinolones et aux aminosides. L'imipenème était efficace sur tous les isolats testés. Dans notre étude, les souches d*'Escherichia* coli étaient sensibles à 100% à l'imipenème. Des résultats similaires étaient observés dans une étude menée au Congoen 2014 [1]. Au Bénin, Hodonou a rapporté en 2016 des résistances de 100% à l'acide-clavulanique des souches d'E coli isolées dans les ISO [7]. En Côte d'Ivoire, Faye-Kette avait rapporté des résultats inferieurs avec 40% de résistance de *E coli* aux inhibiteurs de bétalactamines (l'acide-clavulanique) [3]. Dans notre série, les souches d*'E.col*i présentaient des résistances aux fluoroquinolones et aux aminosides. Des résultats comparables étaient rapportés par les études de Ouédraogo au Burkina Faso [9].

Dans notre étude, les souches de *Klebsiella pneumoniae* étaient sensibles 100% à l'imipenème et à la norfloxacine. Elles étaient résistantes de 75 à 100% aux les bétalactamines. Ces résultats étaient semblables à ceux retrouvés dans des études réalisées au Bénin et en Côte d'Ivoire [3, 7]. Dans notre étude, les souches de *Proteus mirabilis* présentaient des résistances aux betalactamines testés (100% à *l'Amoxicilline,* l'Acide-clavulanique, la ticarcilline, la cefoxitine, la cefalotine et la Céfépime), (50% à la ceftriaxone et la ceftazidine). Ceci est identique aux résultats rapportés par Hodonouménée au Bénin en 2016 [7]. Dans notre étude, les souches de *S.aureus* avaient montré des résistances de 37% et 57% respectivement à la vancomycine et teicoplanine. Ces résultats sont comparables à ceux rapportés par Ouédraogo au Burkina Faso en 2011 [9]. Aussi, la plupart des souches de *S.aureus* avaient montré des résistances de 88,6% à la Pénicilline G, 83% à l'oxacilline. Des résultats proches aux nôtres ont été observés au Burkina Faso et en Côte d'Ivoire [3, 9]. Ces variations de résistance des différentes souches bactériennes isolées vis-à-vis des antibiotiques pourraient s'expliquer par l'absence de stratégies de prescription des antibiotiques, l'automédication, l'ignorance des problèmes de résistance aux antibiotiques sont des causes directes de l'inactivité des bétalactamines aux souches bactériennes isolées. Globalement il était difficile de comparer avec certitude ces résultats à ceux d'autres auteurs vu les différences constatées en rapport avec le choix de molécules testés, l'état pathologique des malades, la pathologie opérée et la flore microbienne de l'hôpital ou du service concerné.

## Conclusion

Les infections du site opératoire (ISO) restent des complications fréquentes de la chirurgie. Il convient de prendre des mesures d'antibioprophylaxies afin de limiter leur survenue.

### Etat des connaissances actuelles sur le sujet

- Les Infections du site opératoire constituent un problème en milieu hospitalier au Niger;
- Les hôpitaux constituent un réservoir des souches multi-résistantes;
- Les mauvaises prescriptions, les traitements probabilistes empiriques, l'automédication, l'utilisation d'antibiotiques de mauvaise qualité sont à la base de la sélection et le maintien des souches multi-résistantes dans les structures de soins.

### Contribution de notre étude à la connaissance

- Profil des germes hospitalier: la présente étude décrit de façon détaillée le profil des germes hospitalier avec leur niveau de résistance vis-à-vis des antibiotiques;
- L'antibioprophylaxie réussie: la présente étude met en exergue la nécessité de bien prescrire les antibiotique mais aussi de prendre en compte les des mesures d'hygiène hospitalière pour éviter la propagation des souches multi-résistantes dans les services de chirurgie;
- Importance de l'Examen Cytobactériologique du pus avec antibiogramme: la présente étude souligne l'importance de l'examen cytobactériologique du pus issu des plaies post opératoire avec antibiogramme avant l'instauration d'un quelconque traitement. Cela permettra d'éviter l'émergence des souches multi-résistantes.

## Conflits d’intérêts

Les auteurs ne déclarent aucun conflit d'intérêts.
